# Auxin biosynthetic key gene *CpYUC10B* in embryo involved in large-fruited Chinese cherry massive abscission during the pit hardening process

**DOI:** 10.3389/fpls.2026.1923869

**Published:** 2026-08-18

**Authors:** Hao Wang, Xiaocheng Liu, Quan Su, Yunfan Yang, Xuping Wu, Jing Zhang, Yan Wang, Wen He, Zhiwei Wu, Mengyao Li, Qing Chen, Ya Luo, Yong Zhang, Haoru Tang, Xiaorong Wang

**Affiliations:** 1 College of Horticulture, Sichuan Agricultural University, Chengdu, Sichuan, China; 2 Key Laboratory of Agricultural Bioinformatics (Ministry of Education), Sichuan Agricultural University, Chengdu, Sichuan, China

**Keywords:** Chinese cherry, fruit abscission, auxin biosynthesis, *CpYUC10B*, function verification

## Abstract

Chinese cherry [*Cerasus pseudocerasus* (Lindl.) G.Don] is an economically important Rosaceae fruit crop in China. Massive fruit abscission in the majority of large-fruited Chinese cherry landraces leads to a catastrophic yield loss, hindering the development of the Chinese cherry industry. Herein, we performed systematic physiological analyses to comprehensively characterize the dynamic changes in auxin-centered phytohormones during fruit development across four Chinese cherry landraces exhibiting varying degrees of fruit abscission. These analyses, along with exogenous indole-3-acetic acid (IAA) rescue experiments, confirmed that the endogenous phytohormone imbalance triggered by reduced IAA accumulation in embryos is the core cause of young fruit abscission. Transcriptomic profiling revealed divergent expression patterns of genes associated with IAA synthesis and transport in the embryo, pedicel, and flesh at two critical fruit developmental stages. Weighted gene co-expression network analysis prioritized the rate-limiting YUCCA flavin monooxygenase encoding gene *CpYUC10B*. Transient overexpression of *CpYUC10B* elevated IAA levels and upregulated polar auxin transporter gene *CpPIN1* expression in embryos, pedicels, and flesh, while repressing the expression of cell wall hydrolase genes in pedicels, consequently reducing fruit abscission. Conversely, VIGS-mediated silencing produced opposite effects. Meanwhile, ectopic overexpression of *CpYUC10B* in tomato delayed distal pedicel abscission after flower removal, accompanied by elevated IAA levels in the abscission zone, upregulated expression of *SlPIN1*, and downregulated expression of cell wall hydrolase genes (*SlEXLB1*, *SlBGL12*, *SlPG*, and *SlPME7*). Collectively, our findings provide a valuable theoretical foundation and practical reference for enhancing yield stability, and offer a new target gene for the genetic improvement of large-fruited cultivars.

## 1 Introduction

Chinese cherry [*Cerasus pseudocerasus* (Lindl.) G. Don], native to China, is one of the commercially important Rosaceae fruit crops (Yü et al., 1986; Zhang et al., 2021). It serves as an excellent economic tree species for developing urban agriculture and rural development. In recent years, frequent extreme weather events have led to massive fruit abscission during the growth and development of Chinese cherry, resulting in significant yield reduction and economic losses. Furthermore, multi-year field investigations have revealed that large-fruited landraces exhibit more severe fruit abscission than small-fruited landraces in this fruit crop. Accordingly, exploring the inducing factors and underlying molecular regulatory mechanisms of fruit abscission in Chinese cherry is of crucial theoretical and practical value for stabilizing high yields, and for the genetic improvement and molecular breeding of new large-fruited Chinese cherry varieties.

Fruit abscission is a programmed cell separation process in the abscission zone (AZ) regulated by endogenous phytohormonal balance (Patterson, 2001; Roberts et al., 2002). Currently, auxin is considered one of the core regulators governing fruit abscission (Kućko et al., 2020; Ma et al., 2024; Zhang et al., 2026). Reduced auxin biosynthesis in embryos or impaired transport toward the AZ can elevate the susceptibility of abscission layer cells to abscission signals, activate cell wall hydrolases, and trigger fruit abscission (Li et al., 2019; Botton et al., 2011; Wang et al., 2024; He et al., 2025). This ultimately results in significant yield loss or even complete crop failure. Therefore, investigating the synthesis and transport of auxin and the expression patterns of auxin-pathway-related genes is crucial for revealing the mechanism underlying fruit abscission.

Generally, *de novo* auxin biosynthesis can be classified into Trp-dependent and Trp-independent pathways, with the former being extensively studied (Solanki and Shukla, 2023). In the Trp-dependent pathway, the indole-3-pyruvate (IPA) pathway is the predominant route for primary IAA biosynthesis in plants (Morffy and Strader, 2020). The content and distribution of auxin in various tissues are co-regulated by a series of auxin biosynthesis-related (e.g., *TAR*, *YUCCA*), transport-related (e.g., *PIN1*), and metabolism-related genes (e.g., *DAO*, *GH3*). Recent studies have revealed striking transcriptional differences in auxin-pathway-related genes before and after abscission initiation, implying that these genes may participate in the regulatory network of abscission development (Larson et al., 2021; Guo et al., 2024; Wang et al., 2024; Hou et al., 2025). Particularly, the expression patterns of *YUCCA* (*YUC*) genes that encode rate-limiting enzymes in auxin synthesis can remarkably influence endogenous IAA levels, which exacerbate or alleviate organ abscission (Casanova-Sáez et al., 2021; Qiu et al., 2021; Sato et al., 2022; Luo and Di, 2023; Li et al., 2023). This has been well supported by the loss-of-function mutations of *YUC* genes in model plants (Cheng et al., 2006; Meng et al., 2023). Collectively, auxin-pathway-related genes play crucial regulatory roles in organ abscission.

Currently, although significant progress has been made in the molecular mechanism of fruit abscission, and the crucial roles of auxin-pathway-related genes have been highlighted in governing abscission zone development and physiological fruit abscission in many other fruit crops, this mechanism still remains uncharacterized in Chinese cherry. Therefore, in this study, to unravel the molecular mechanism underlying auxin-mediated fruit abscission in Chinese cherry, we conducted a comparative analysis of four Chinese cherry landraces with distinct abscission phenotypes. Our objectives are to (i) characterize stage-specific abscission dynamics in relation to embryo development and endogenous phytohormones, (ii) identify auxin-related candidate genes, and (iii) functionally validate key genes using transient overexpression, virus-induced gene silencing (VIGS) in Chinese cherry, and heterologous stable transformation in tomato. This study provides a mechanistic foundation for understanding auxin-regulated fruit abscission, with broad implications for Chinese cherry crop improvement.

## 2 Materials and methods

### 2.1 Plant materials

Four Chinese cherry landraces with distinct abscission phenotypes were used in this study, LQ (severe abscission) and HF, NH631, and BJ7-2 (low abscission). All materials were grown under field conditions at the Cherry Germplasm Repository of Sichuan Province, China. Five trees exhibiting uniform growth vigor were selected for each landrace. During fruit development stages S1 to S5 (S1, DAF15; S2, DAF21; S3, DAF27; S4, DAF42 (DAF33 for NH631); S5, DAF48 (DAF42 for NH631)), samples of pedicels, embryos, and flesh were collected separately from abscised and normal fruits, immediately frozen in liquid nitrogen, and stored at -80 °C.

### 2.2 Observation of fruit abscission rate and embryo abortion rate

From each tree, one branch was selected from each cardinal direction (east, west, south, and north), tagged, and used to record the number of flowers at full bloom. Fruit retention and abscission data were collected at the first physiological fruit abscission stage (S1), pre-fruit pit hardening stage (S2), pit hardening stage (S3), color transition and swelling stage (S4), and ripening stage (S5). The stage-specific abscission rate (%) was calculated as (number of fruits abscised at the stage/initial number of fruits) × 100. Embryos were dissected from both abscised and persisting fruitlets at each stage, and the embryo abortion rate (%) was calculated as (number of aborted seeds/total number of seeds) × 100.

### 2.3 Measurement of pollen germination rate

Pollen germination assays were performed *in vitro* following established protocols with minor modifications (Jiang et al., 2006). Fresh pollen grains were evenly spread on solid germination medium containing 0.01% (w/v) boric acid, 10% (w/v) sucrose, and 1% (w/v) agar, and then incubated at 23 °C in darkness for 4–6 h. A pollen grain was considered germinated when the pollen tube length exceeded the grain diameter. For each genotype, three independent replicates were examined, with at least 50 pollen grains counted per field of view. The germination rate was calculated as the percentage of germinated pollen grains.

### 2.4 HPLC determination of endogenous phytohormones

For the extraction of IAA and ABA, 1.0 g of powdered sample was mixed with 10 mL of cold 80% methanol (stored at 4°C) and extracted in the dark for 16 h. After centrifugation (6000 rpm, 10 min), the supernatant was collected, and the pellet was re-extracted twice with 80% cold methanol (1:5, v/v). The combined supernatants were treated with 0.2 g of polyvinylpolypyrrolidone (PVPP), shaken for 20 min, then filtered and evaporated under vacuum at 40 °C. The resulting residue was dissolved in 10 mL of phosphate buffer (pH 3.5) and partitioned three times using an equal volume of ethyl acetate. High-performance liquid chromatography (HPLC) analysis was performed using a Thermo U3000 system equipped with a Syncronis C18 column (250 mm × 4.6 mm, 5 μm) at 35°C. The mobile phase consisted of methanol (phase A) and 0.1% acetic acid (pH 3.2, phase B) at a ratio of 55:45 (v/v). The flow rate was set at 1.0 mL/min, the detection wavelength at 254 nm, and the injection volume at 10 μL. Phytohormone quantification was conducted using the external standard method.

### 2.5 Exogenous IAA treatment of Chinese cherry LQ

Three consecutive applications of IAA solution (30 μg·mL^-1^) were made to LQ fruits during the S2 stage (DAF18, 21, and 24). Water-sprayed controls (CK) were established and used for comparison. Three independent biological replicates per treatment were monitored for fruit retention at DAF18, 24, 36, 45, and 48, allowing for the calculation of stage-specific (S2, S3, S4 and S5) abscission rates and final fruit set.

### 2.6 RNA extraction and RT-qPCR analysis

Total RNA was extracted using the RNAprep Pure Plant Plus Kit (Tiangen Biotech, Beijing, China), and quality was assessed by 1% agarose gel electrophoresis and Nanodrop spectrophotometry (Wang et al., 2023). mRNA was enriched using Oligo(dT)-conjugated magnetic beads and reverse-transcribed into cDNA. RT-qPCR was performed using SYBR Green I Supermix (TransGen) with gene-specific primers (**Supplementary Table 1**). Relative expression was calculated by the 2^−ΔΔCT^ method, *SlGAPDH* and *SlActin* served as reference genes for tomato (*Solanum lycopersicum* L.), while *CpGAPDH* and *CpTIF5A* were used for Chinese cherry (*Cerasus pseudocerasus* (Lindl.) G.Don). Three independent biological replicates, each with three technical replicates, were analyzed.

### 2.7 Transcriptome sequencing and WGCNA

Transcriptome sequencing of 27 samples — embryos, flesh, pedicels from normal fruits at DAF18 (18N), normal fruits at DAF 27 (27N), and abscised fruits at DAF 27 (27A), with three replicates each — was performed on the Illumina NovaSeq 6000 platform. Clean reads were aligned to the Chinese cherry reference genome (CNP0006741) using Hisat2 software (v2.1.0). Gene expression levels were quantified as fragments per kilobase of transcript per million mapped reads (FPKM). Differential expression genes (DEGs) were identified using DESeq2 with the criteria |log_2_FC| ≥ 2 and *p* ≤ 0.05 (Love et al., 2014). Gene Ontology (GO) and Kyoto Encyclopedia of Genes and Genomes (KEGG) enrichment analyses were conducted using clusterProfiler (v3.18.1). Weighted gene co-expression network analysis (WGCNA, v1.66) was applied to construct an unsigned co-expression network using the “blockwiseModules” function. The IAA, ABA, and IAA/ABA ratio data used for WGCNA were obtained from the same batch of fruit samples as those used for RNA-seq. Sequencing data have been deposited in the Life Big Data Platform of China National GeneBank (CNGBdb) under accession number CNP0008745.

### 2.8 Subcellular localization of *CpYUC10B*

For subcellular localization analysis, the coding sequence of *CpYUC10B* was cloned into the pCAMBIA1305-GFP vector to generate a C-terminal GFP fusion construct. The recombinant plasmid was co-infiltrated with an endoplasmic reticulum-localized mCherry marker into *Nicotiana benthamiana* leaves via *Agrobacterium*-mediated transient expression. Three days post-infiltration, GFP and mCherry fluorescence signals were visualized using an FV3000 confocal laser scanning microscope (Olympus, Japan).

### 2.9 Transient overexpression and virus-induced gene silencing of *CpYUC10B*

The full-length coding sequence (CDS) of *CpYUC10B* was cloned into the pCAMBIA1305 vector, and a 300-bp fragment was reverse-inserted into the pTRV2 vector. The recombinant plasmids were transformed into *Agrobacterium tumefaciens* strain GV3101. For VIGS, *Agrobacterium tumefaciens* strains harboring pTRV1 and pTRV2-*CpYUC10B* were cultured separately in LB medium supplemented with appropriate antibiotics at 28°C to an OD_600_ of 0.7~0.8. The cells were harvested, resuspended in infiltration buffer (10 mM MgCl_2_, 10 mM MES, and 250 μM acetosyringone, pH 5.6) to a final OD_600_ of 0.7~0.8, and then the pTRV1 and pTRV2-*CpYUC10B* suspensions were mixed at a 1:1 ratio (v/v) and incubated at room temperature for 3~4 h before infiltration. The control was prepared by mixing pTRV1 with an empty pTRV2 vector following the same procedure.

Chinese cherry fruits at stage S2 (DAF18) were infiltrated with approximately 200 μL per fruit until visible tissue permeation was achieved. After infiltration, the fruits were kept in darkness for 48 h and then transferred to standard growth conditions. Abscission rates were recorded every 3 days from 7 dpi until fruit maturity. At the S3 stage (12 dpi), treated fruits were harvested for abscission-related trait analysis. For each assay, 40 fruits from the same tree were treated. Three biological replicates (n = 40 fruits per tree, one tree per replicate) were performed.

### 2.10 Genetic transformation of the *CpYUC10B* in tomato

Wild-type tomato (*Solanum lycopersicum* cv. ‘Micro Tom’) was used as the recipient material. The above vector was transformed into tomato explants via Agrobacterium-mediated transformation to generate transgenic plants (Pozueta-Romero et al., 2001). Subsequently, leaves were sampled for genomic DNA and total RNA extraction to conduct positive transgenic line identification and gene expression quantification. Finally, three transgenic lines with the highest target gene expression levels were selected for subsequent functional experiments.

### 2.11 Data analysis

Data were analyzed using GraphPad Prism (Version 10.1.2) and Microsoft Excel 2021. Differences among multiple groups were evaluated by one-way analysis of variance, with *post-hoc* pairwise comparisons conducted via Tukey’s HSD test. Comparisons between two groups were performed using independent Student’s t-test. Statistical significance was set at *P* < 0.05.

## 3 Results

### 3.1 Embryo abortion is a key factor contributing to the massive fruit abscission at pit hardening stage

**Figure 1:**
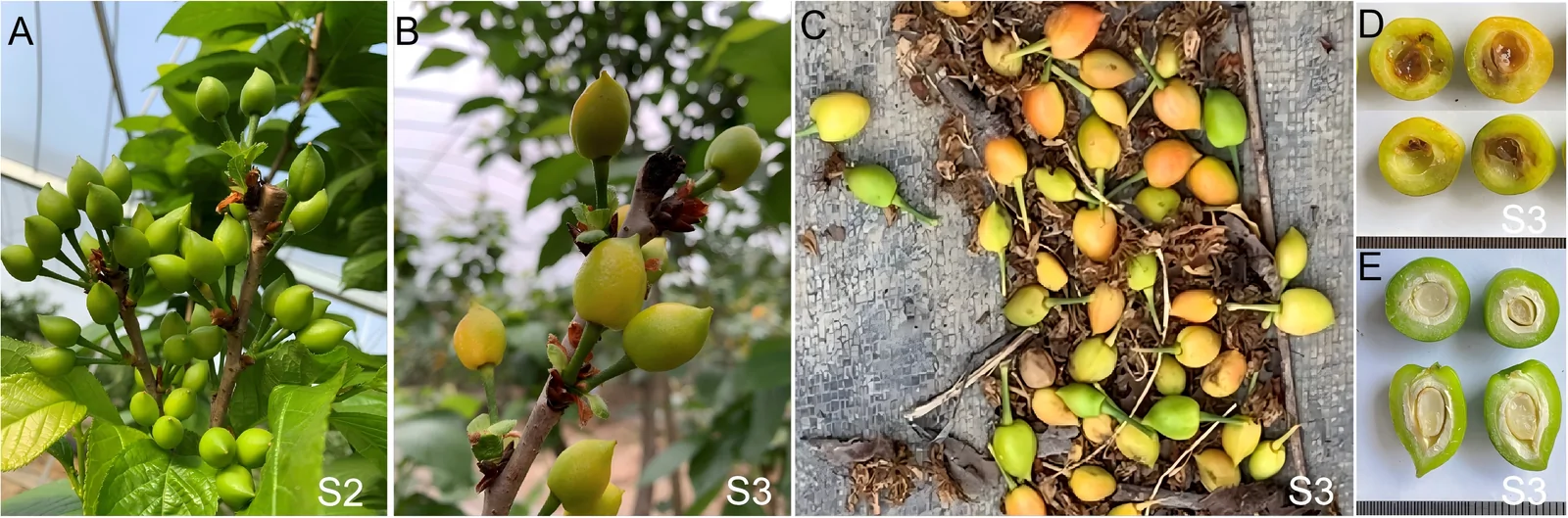
Fruit abscission dynamics and embryo development in Chinese cherry (LQ). **(A)** Normal fruit development and fruit set at stage S2. **(B)** Fruits at stage S3, immediately prior to abscission. **(C)** Abscised fruits at stage S3. **(D)** Aborted embryos in abscised fruits at stage S3. **(E)** Embryo phenotype of normal fruits at stage S3.

To clarify fruit abscission dynamics, fruit development in four Chinese cherry landraces was divided into five stages (S1–S5) based on morphological changes (**Supplementary Figure 1**). Throughout S2–S5, most landraces exhibited stage-specific fruit abscission, peaking at S3 stage (7.15%–69.29%) (**Table 1**, **Figures 1A–C**). Among these landraces, LQ showed significantly higher abscission than the other three landraces (HF, BJ7-2, and NH631), with a total abscission rate of 94.75% and a final fruit set rate of only 5.25% at maturation (**Table 1**). Collectively, peak abscission at S3 is a primary determinant of LQ yield limitation.

**Table 1:** The fruit abscission rates and productivity of four Chinese cherry landraces.

Landraces fruit weight (g)	Flower/fruit (n)	Abscission rate (%)				Fruit set (%)	Embry abortion (%)	Yield
	S1	S2	S3	S4+S5	Total			
LQ(>6)	727/397	11.59 ± 1.59a	69.29 ± 3.00a	13.87 ± 4.26a	94.75 ± 5.03a	5.25 ± 5.03d	97.14 ± 2.74a	Poor
HF(4.0-5.0)	249/159	7.35 ± 1.65b	31.05 ± 3.28b	7.83 ± 2.37b	46.23 ± 2.68b	53.77 ± 2.68c	78.37 ± 1.08b	Good
BJ7-2(<3.0)	339/298	14.25 ± 3.26a	13.01 ± 2.24c	5.1 ± 2.71bc	32.36 ± 5.76c	67.64 ± 5.76b	46.30 ± 4.57c	Very good
NH631(<3.0)	1873/990	1.93 ± 0.25c	7.15 ± 1.31d	0.98 ± 0.22c	10.07 ± 1.34d	89.93 ± 1.34a	42.57 ± 0.88c	Very good

Data are presented as means ± standard deviation. Lowercase letters indicate significant differences at the 0.05 level.

To investigate the underlying causes of massive fruit abscission in LQ, we examined stigma and another development, both of which were normal (**Supplementary Figures 2A–D**). Meanwhile, the pollen germination rate (60%) showed no significant difference between LQ and high-yielding landraces (BJ7–2 and NH631), ruling out abnormal floral organ development as a cause of massive fruit abscission at S3 (**Supplementary Figure 2E**).

Furthermore, anatomical observations revealed that embryos were aborted in all abscised fruits but were intact in normally developed fruits (**Figures 1D, E**). The embryo abortion rate of LQ (97.14%) was significantly higher than that of BJ7-2, HF, and NH631 (**Table 1**). Regression analysis confirmed a strong positive correlation between fruit abscission rate and embryo abortion rate (R²=0.87, **Supplementary Figure 3**). These results indicate that embryo abortion is closely associated with fruit abscission. Accordingly, subsequent research focused on elucidating the molecular regulatory mechanisms underlying abnormal fruit abscission at the pit hardening stage (S3).

### 3.2 Massive fruit abscission in LQ at the S3 stage is closely correlated with reduced IAA levels in embryos

**Figure 2:**
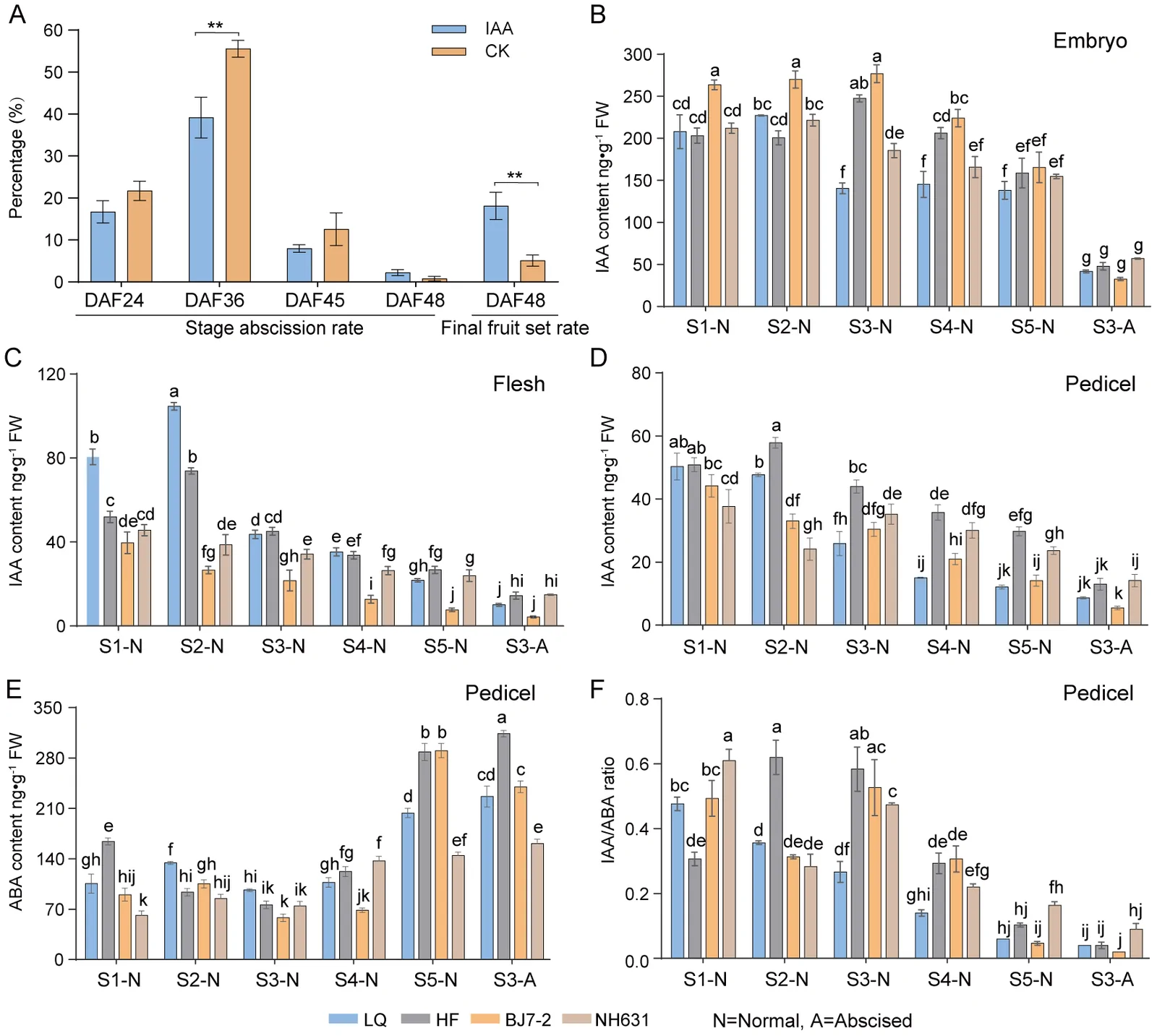
Exogenous IAA treatment on LQ fruits and dynamic analysis of IAA and ABA contents in representative landraces during fruit development. **(A)** Fruit set and abscission dynamics in LQ fruits under exogenous IAA treatment. CK, Water-sprayed controls. **(B-D)** IAA levels in the embryos, flesh, and pedicels of normal and abscised fruits from LQ, HF, BJ7-2, and NH631. **(E)** ABA levels and **(F)** IAA/ABA ratio in the pedicels of normal and abscised fruits from LQ, HF, BJ7-2, and NH631. Student’s t-test was used for **Figure 2A**, while Tukey’s HSD test was applied for **(B-F)**. Different lowercase letters indicate significant differences at the 0.05 level in **(B-F)**; *, *P* < 0.05, **, *P* < 0.01, ***, *P* < 0.001.

The embryo serves as the primary source of IAA, embryo abortion impairs IAA synthesis, thereby leading to reduced IAA levels, disrupted phytohormonal balance, and ultimately fruit abscission (Racsko et al., 2007). To verify the potential regulatory role of auxin in Chinese cherry fruit abscission, exogenous IAA was applied to LQ, and fruit abscission rates at different developmental stages and the fruit set rate at maturity were investigated (**Figure 2A**). Compared with control, IAA treatment significantly reduced fruit abscission during S3 (DAF24–DAF36) and increased the fruit set rate at maturity (**Figure 2A**), effectively alleviating the massive fruit abscission in LQ at the pit hardening stage (S3).

To further investigate phytohormonal dynamics, we quantified endogenous IAA in the embryo, flesh, and pedicel of normally developing fruits at S1–S5 and abscised fruits at S3 from four landraces. Pedicel ABA contents and IAA/ABA ratios in pedicel were also determined. At the same developmental stage, embryo IAA levels were 5 ~ 6-fold higher than those in pedicels and flesh (**Figures 2B–D**). At the peak abscission stage (S3), IAA levels in all tissues of abscised fruits were significantly lower than those of normal fruits across all landraces, with the most pronounced reduction in embryos (**Figure 2B**). At S3, ABA content in pedicel of abscised fruits increased by 2.16 ~ 4.13-fold compared with those of normal fruits (**Figure 2E**), while IAA/ABA ratios were significantly lower in abscised fruits than in normal fruits (**Figure 2F**).

In summary, IAA deficiency was universally associated with fruit abscission among different genotypes. Insufficient auxin, particularly in embryos, is a critical factor triggering fruit abscission at S3. The massive fruit abscission in LQ may result from the reduced IAA production in embryo and the consequently decreased IAA/ABA ratio in pedicel.

### 3.3 RNA-seq and GO enrichment analysis of differentially expressed genes

**Figure 3:**
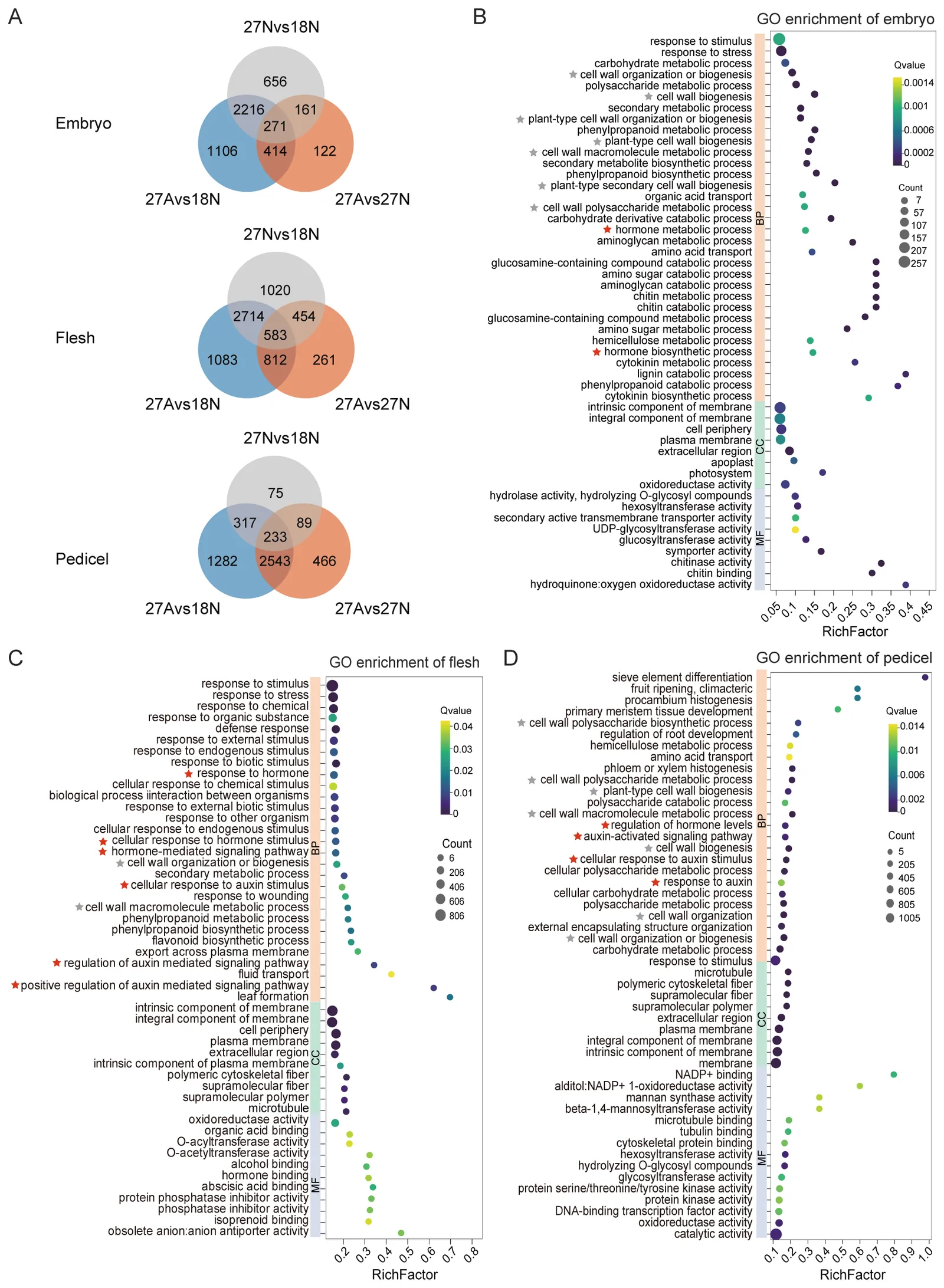
Venn diagrams and GO enrichment analysis of DEGs within different tissues. **(A)** Venn diagrams of DEGs within different tissues. **(B)** GO enrichment analysis in embryo. **(C)** GO enrichment analysis in flesh. **(D)** GO enrichment analysis in pedicel. Red stars represent GO terms associated with plant hormone biosynthesis, metabolism, and regulation, while gray stars represent GO terms related to cell wall. All displayed GO terms are statistically significant with Q-value < 0.05, and the top 50 terms were then selected for plotting.

To elucidate the causes underlying auxin deficiency and the signaling pathways that trigger abscission at the molecular level, we performed comparative transcriptome profiling of flesh, pedicel and embryo from normal and abscised fruits at DAF18 (S2) and DAF27 (S3). Then, the comparative transcriptomic analyses were conducted to identify the differentially expressed genes (DEGs) within each tissue (**Figure 3A**) and conducted Gene Ontology (GO) analyses after excluding DEGs unique to the 18N vs 27N comparison.

The GO enrichment analysis for DEGs across flesh, pedicel, and embryo was categorized into three main ontologies (**Figures 3B–D**). Within the biological process (BP) category, significantly enriched terms included hormone metabolic processes, hormone-mediated signaling pathways, and responses to hormone stimuli. For the cellular component (CC) ontology, major enriched terms included plasma membrane, cell periphery, and extracellular region. In the molecular function (MF) category, terms with the highest number of assigned genes were oxidoreductase activity and catalytic activity. Notably, DEGs in the embryo were enriched in hormone metabolic and biosynthetic processes, whereas those in the pedicel abscission zone (AZ) were enriched in auxin response and cell wall organization pathways. These results indicate that hormone-mediated transcriptional regulation may play a critical role in fruit abscission.

### 3.4 Weighted gene co-expression network analysis and candidate gene identification

**Figure 4:**
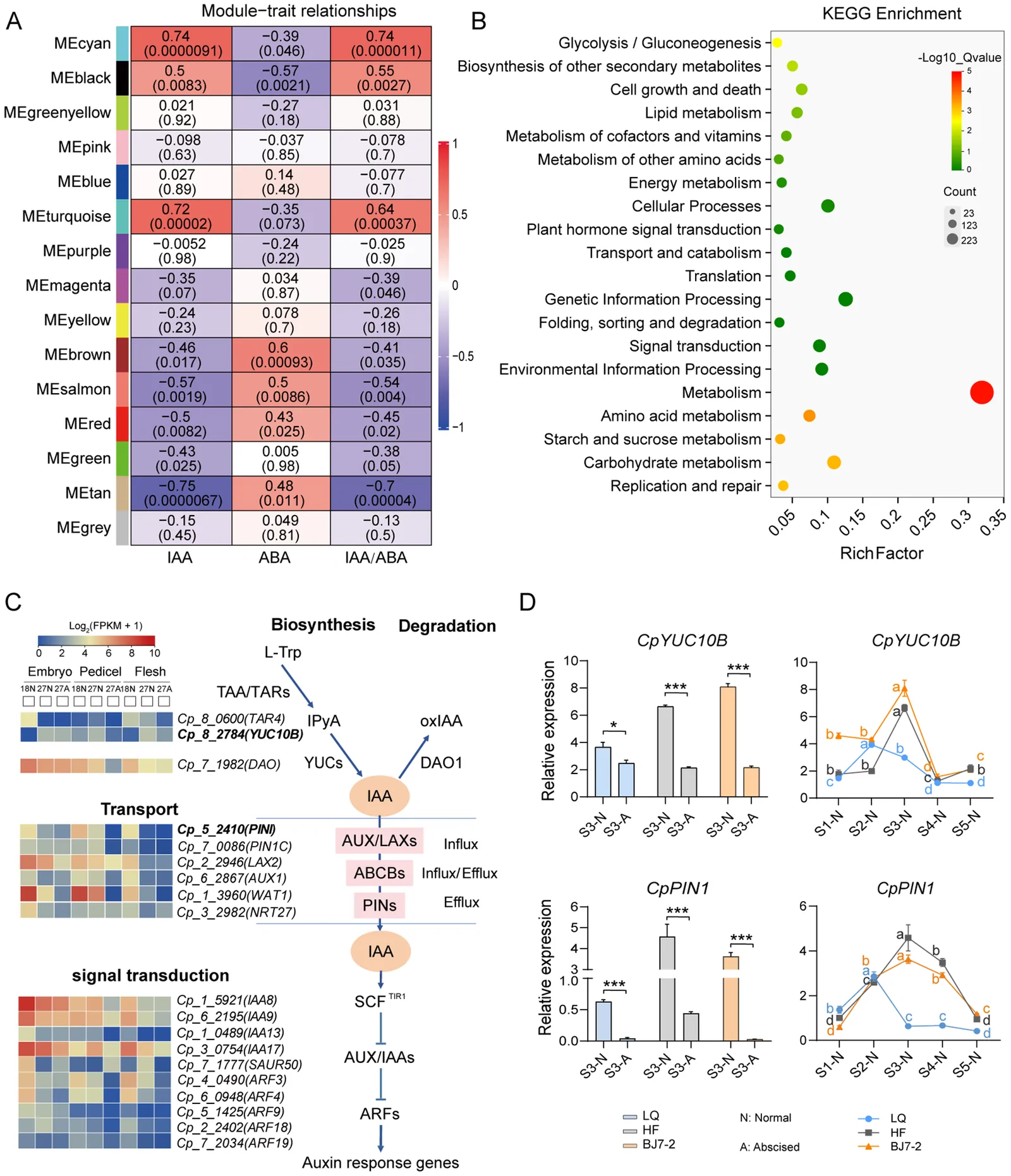
WGCNA for identifying the auxin-related potential genes. **(A)** Module-trait correlations. In each cell, the top number represents the correlation coefficient (R), and the value in parentheses represents the *P* value. The color scale indicates module-trait correlations ranging from -1 (purple) to 1 (red). **(B)** KEGG enrichment analysis of turquoise and cyan module genes. **(C)** DEGs associated with auxin biosynthesis, transport, metabolism, and signal transduction identified in the turquoise and cyan modules. The color scale indicates log_2_(FPKM + 1) values. **(D)** Expression pattern of *CpPIN1* and *CpYUC10B* in the embryos of normal and abscised fruits from LQ, HF, and BJ7–2 at various developmental stages by RT-qPCR. Statistical significance analysis was performed only within each landrace for **(D)**, lowercase letters indicates significant differences at 0.05 level. *, *P* < 0.05, **, *P* < 0.01, ***, *P* < 0.001.

To further elucidate the role of auxin in regulating fruit abscission in Chinese cherry, we performed WGCNA by integrating transcriptomic data and phytohormone profiles. After filtering 8313 DEGs, 15 co-expression modules were generated using 6801 of these genes. The MEturquoise and MEcyan modules showed significant positive correlations with IAA content (R = 0.72 and 0.74, respectively) and the IAA/ABA ratio (R = 0.64 and 0.74, respectively) (**Figure 4A**). KEGG enrichment analysis indicated that the 2512 genes from these two modules were predominantly enriched in pathways involving amino acid metabolism, carbohydrate metabolism, starch and sucrose metabolism, glycolysis/gluconeogenesis, signal transduction, plant hormone signal transduction, and cell growth and death (**Figure 4B**), including two auxin biosynthesis-related (*CpTAR4*, *CpYUC10B*), one auxin degradation-related (*CpDAO*), five auxin transport-related (*CpPIN1*, *CpLAX2*, etc.), and ten signal transduction-related genes (*CpIAA8*, *CpSAUR50*, *CpARF3*, etc.) (**Figure 4C**, **Supplementary Table 2**). These results suggest that these modules may participate in the regulation of Chinese cherry fruit abscission through the coordinated modulation of auxin synthesis, metabolism, and signal transduction.

The expression dynamics of auxin biosynthesis genes *CpYUC10B* and polar transport gene *CpPIN1* were further comparatively analyzed in embryos of the severe-abscission landrace LQ and the low-abscission landraces HF and BJ7-2, as well as in normal (N) and abscised (A) embryos at the S3 stage. RT-qPCR analyses showed that *CpYUC10B* and *CpPIN1* were significantly downregulated in LQ at the S3 stage but remained stable or upregulated in HF and BJ7-2 (**Figure 4D**). The expression levels of the two genes were significantly lower in abscised embryos than in normal embryos at the S3 stage (**Figure 4D**). These findings suggest that the auxin biosynthesis key rate-limiting enzyme gene *CpYUC10B* and the polar transport gene *CpPIN1* may play potential key regulatory roles in fruit abscission of Chinese cherry.

### 3.5 Characterization and functional validation of *CpYUC10B* in Chinese cherry fruit abscission

**Figure 5:**
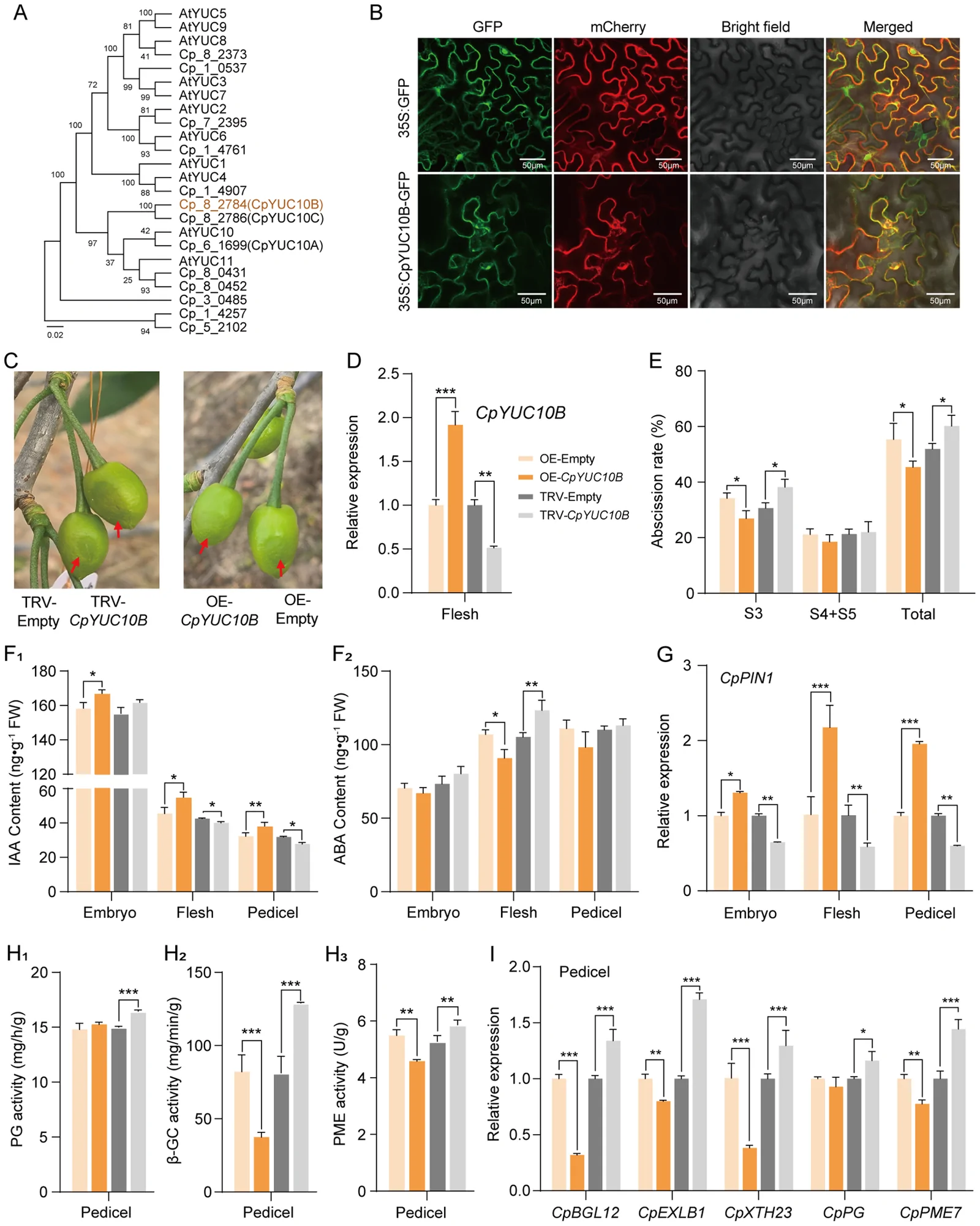
Functional verification of *CpYUC10B* in Chinese cherry fruits. **(A)** Phylogenetic analysis of CpYUC10B from Chinese cherry and AtYUCs from *Arabidopsis thaliana*. The tree was constructed using the Neighbor-Joining (NJ) method. **(B)** Subcellular localization of CpYUC10B. ER-mCherry was used as the ER marker. Scale bar = 50 μm. **(C)** Phenotypes of overexpression and silencing of *CpYUC10B* cherry fruits after infiltration. The arrows indicate infiltration sites. **(D)** Relative expression level of *CpYUC10B*. **(E)** Fruit abscission rate. **(F_1_, F_2_)** IAA and ABA content. **(G)** Relative expression level of *CpPIN1*. **(H_1_–H_3_)** Cell wall hydrolase activity of Polygalacturonase (PG), β-Glucosidase (β-GC) and Pectinesterase (PME). **(I)** Expression profiles of cell wall hydrolase genes in response to *CpYUC10B* overexpression and silencing in Chinese cherry. *P < 0.05, **P < 0.01, ***P < 0.001.

To determine the molecular characteristics and evolutionary relationship of *CpYUC10B*, we performed sequence alignment and phylogenetic analyses. The results showed that *CpYUC10B* encodes a 385-amino acid protein containing the conserved flavin monooxygenase (FMO) domain characteristic of the YUC family, and exhibited high sequence conservation across species (**Figure 5A**, **Supplementary Figure 4**). Additionally, subcellular localization assays revealed that CpYUC10B localized to the endoplasmic reticulum (**Figure 5B**).

To investigate the role of *CpYUC10B* in fruit abscission, we performed transient overexpression and VIGS in Chinese cherry fruits (**Figure 5C**). Compared with the empty vector control, *CpYUC10B* transcript levels were upregulated 1.92-fold in overexpressed fruits and downregulated 1.95-fold in *CpYUC10B*-silenced fruits (**Figure 5D**), confirming successful genetic transformation.

Phenotypically, the abscission rate of *CpYUC10B*-overexpressed fruits was significantly lower than that of the OE-empty, whereas much higher abscission rate was observed in *CpYUC10B*-silenced fruits than in TRV-empty (**Figure 5E**). Concurrently, *CpYUC10B* overexpression significantly elevated IAA levels in embryos, flesh, and pedicels (**Figure 5F1**), while significant changes of ABA contents were only observed in flesh (**Figure 5F2**). RT-qPCR further revealed that *CpPIN1* was significantly upregulated in flesh, pedicels, and embryos. Conversely, *CpYUC10B*-silenced significantly reduced IAA levels in flesh and pedicels (**Figure 5F1**), elevated ABA levels in flesh (**Figure 5F2**), and increased cell wall hydrolase activities (β-GC and PME) in the pedicel (**Figure 5H2, H3**).

Further analysis of the pedicel revealed that *CpYUC10B* overexpression markedly suppressed the expression levels of cell wall hydrolase genes, including *CpEXLB1*, *CpBGL12*, *CpXTH23*, and *CpPME7* (**Figure 5I**). Taken together, these findings suggest that *CpYUC10B*, as a rate-limiting enzyme in auxin biosynthesis, modulates not only auxin content but also polar auxin transport, thereby regulating fruit abscission.

### 3.6 *CpYUC10B* overexpression delays tomato pedicel abscission by modulating auxin levels and cell wall metabolism-related gene expression

**Figure 6:**
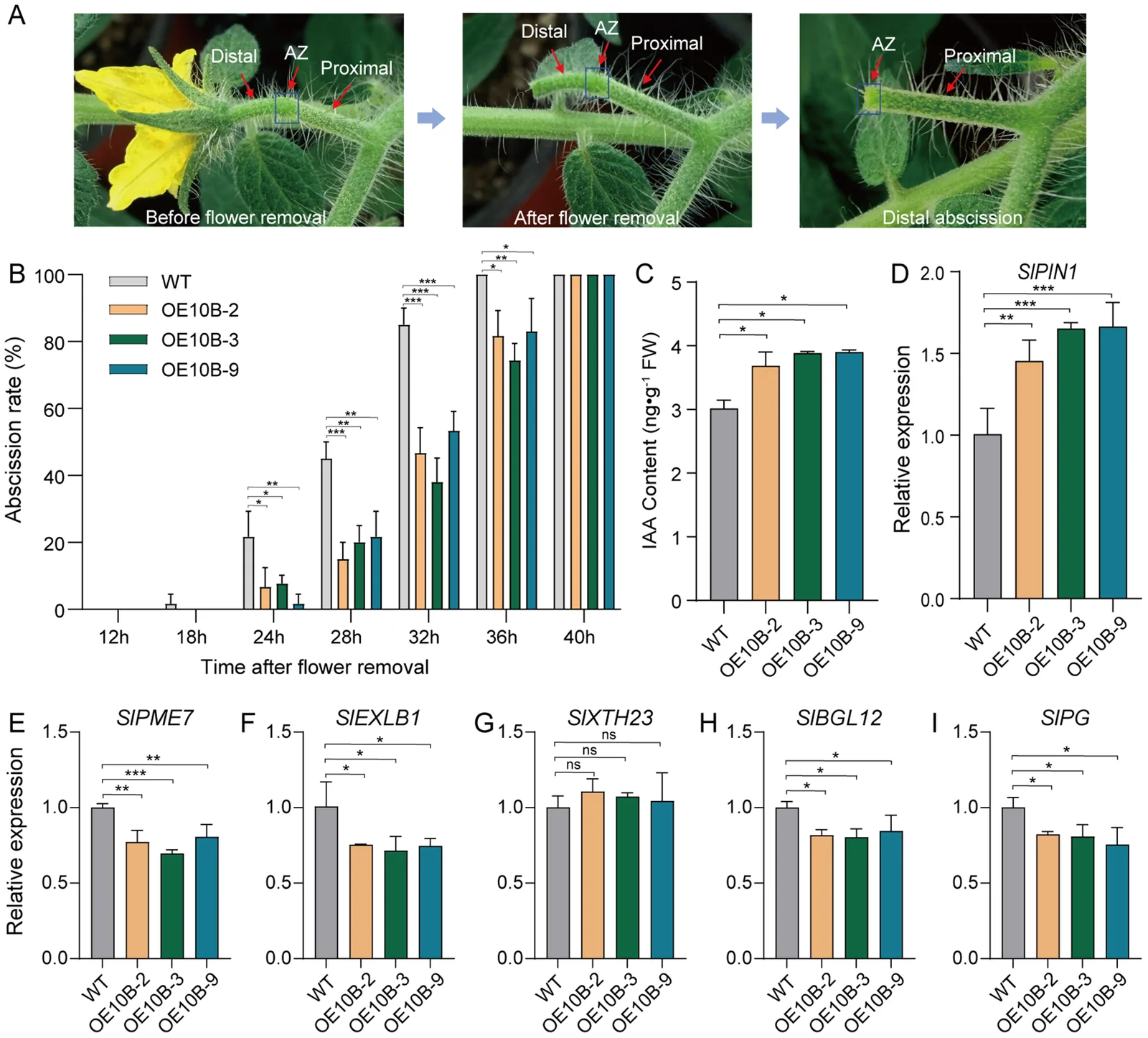
*CpYUC10B* overexpression delays tomato distal pedicel abscission by modulating auxin levels and cell wall metabolism-related gene expression. **(A)** Experiment of flower removal treatment in tomato. **(B)** Investigation of distal pedicel abscission rate and abscission time in *CpYUC10B*-overexpressing (OE-10B) and wild-type (WT) tomato. Data are mean ± SEM (n = 3 biological replicates; 100 pedicels per replicate). *, *P* < 0.05, **, *P* < 0.01, ***, *P* < 0.001, two-tailed Student’s t-test. **(C)** Auxin levels in the abscission zone (AZ) of OE-10B and WT tomato pedicels at 18 h post-flower removal. **(D)** Expression level of the polar auxin transport gene *SlPIN1* in the pedicel AZ of OE-10B and WT tomato at 18 h after flower removal. **(E–I)** RT-qPCR analysis of cell wall hydrolase genes in the pedicel AZ of OE and WT tomato at 18 h after flower removal. The genes examined included pectin methylesterase (*SlPME7*), expansin (*SlEXLB1*), xyloglucan endotransglucosylase/hydrolase (*SlXTH23*), β-glucosidase (*SlBGL12*), and polygalacturonase (*SlPG*). For IAA content and gene expression assays (**Figures 6C–I**), data are mean ± SEM, n = 3 biological replicates, each comprising three technical replicates. *, *P* < 0.05, **, *P* < 0.01, ***, *P* < 0.001. two-tailed Student’s t-test.

To further verify the regulatory function of *CpYUC10B*, overexpression experiments were conducted in tomato (*Solanum lycopersicum* L.). After confirming the positive transgenic lines via PCR and characterizing their *CpYUC10B* expression levels by qRT-PCR (**Supplementary Figures 5A, B**), three high-expression lines (OE10B-2, OE10B-3, and OE10B-9) and the wild-type (WT) were selected as experimental materials. Flowers at the same developmental stage were treated by removing the flower, leaving only the distal pedicel, and the natural abscission process was monitored by recording the distal pedicel abscission rate (**Figure 6A**).

Phenotypic observation showed that distal pedicel abscission began at 18 h after flower removal in the WT plants and full abscission was achieved by 36 h, whereas compared with the WT, the abscission in the *CpYUC10B*-overexpressed lines initiated at 24 h and was fully completed by 40 h, representing delays of 6 h and 4 h in abscission initiation and completion, respectively (**Figure 6B**). Furthermore, at the same time points after flower removal, the distal pedicel abscission rates in the overexpression lines were significantly lower than those in the WT, indicating that *CpYUC10B* overexpression delayed pedicel abscission.

Further analysis at 18 h after flower removal revealed that IAA levels in the abscission zone (AZ) were significantly higher in the overexpression lines than in the WT (**Figure 6C**). RT-qPCR showed that the polar auxin transport gene *SlPIN1* was significantly upregulated in the overexpression lines (**Figure 6D**), while the expression of *SlPME7*, *SlEXLB1*, *SlBGL12*, and *SlPG* was significantly downregulated, except for *SlXTH23*, with no significant difference in expression level (**Figures 6E–I**). Collectively, these findings indicate that *CpYUC10B* can delay pedicel abscission by elevating IAA levels and modulating cell wall hydrolase gene expression in the AZ.

## 4 Discussion

### 4.1 Embryo-derived auxin deficiency as the primary driver of stage-specific fruit abscission in Chinese cherry

The present study demonstrates that fruit abscission in Chinese cherry is highly stage-specific, peaking at the pit hardening stage (S3), and tightly associated with embryo abortion (**Table 1**, **Supplementary Figure 3**). These observations are consistent with previous reports in sweet cherry (Qiu et al., 2021) and avocado (Garner and Lovatt, 2016). As a key developmental transition stage for drupes, the pit hardening stage (S3) is defined by endocarp lignification, cessation of mesocarp growth, and elevated metabolic requirements for embryonic development (Fenn and Giovannoni, 2020).

Spatio-temporal analysis of endogenous indole-3-acetic acid (IAA) revealed significantly higher IAA accumulation in embryos than in fruit flesh and pedicels, identifying the embryo as the primary site of IAA synthesis and accumulation in Chinese cherry fruits (**Figure 2B**). In comparison, all tissues of abscised fruits exhibited pronounced IAA depletion. The classic auxin gradient theory states that elevated auxin levels sustain the quiescent state of the abscission zone (AZ) through polar auxin transport (Addicott and Lynch, 1955). A compromised auxin gradient can increase the susceptibility of AZ cells to abscission signals (Abeles and Rubinstein, 1964; Taylor and Whitelaw, 2001; Ma et al., 2021). This has also been observed in our study, where exogenous IAA application effectively alleviated fruit abscission in the LQ landrace and improved mature fruit set (**Figure 2A**). Similar auxin-mediated rescue effects have been documented in citrus, litchi and other woody crops (Peng et al., 2013; Xie et al., 2018; Liu et al., 2026).

Taken together, IAA deficiency may act as an initial trigger for fruit abscission at S3 stage in Chinese cherry. Reduced auxin gradient and lowered IAA/ABA ratio in the AZ enhance the sensitivity of AZ cells to abscission signals, consequently activating the abscission program (Taylor and Whitelaw, 2001; Ma et al., 2021). Furthermore, defective auxin transport has been proven to promote ABA biosynthesis (Wilmowicz et al., 2016; Kućko et al., 2020). ABA-dependent abscission regulation relies on extensive phytohormonal crosstalk (Estornell et al., 2013). In this study, our results showed markedly higher ABA levels in abscised fruits than in normally developing fruits, verifying the positive involvement of ABA in Chinese cherry fruit abscission.

In summary, embryo-derived IAA insufficiency causes IAA depletion in the AZ, which disturbs endogenous hormonal homeostasis, accelerates ABA accumulation. This disturbed hormonal homeostasis is involved in the initiation of fruit abscission. Nevertheless, the precise regulatory mechanisms underlying signal integration and sophisticated hormonal crosstalk in this process remain to be fully elucidated.

### 4.2 *CpYUC10B* as a rate-limiting node in auxin-mediated abscission control

As a key phytohormone, auxin plays a pivotal role in regulating plant organ abscission (Meir et al., 2010). Transcriptome-WGCNA integration identified *CpYUC10B*, which encodes a YUCCA flavin monooxygenase, as the strongest auxin-biosynthetic candidate correlated with embryonic IAA content. As a rate-limiting enzyme in auxin biosynthesis, *CpYUC10B* is positioned at a critical control point where small expression changes can be amplified into significant physiological consequences (Zhao, 2010; Mashiguchi et al., 2011).

The role of *CpYUC10B* in delaying abscission was validated by its ectopic overexpression in tomato (delaying abscission initiation and completion by 6 h and 4 h, respectively), and this phenotypic evidence was corroborated by molecular and physiological data (**Figures 6C–I**). Meanwhile, transient overexpression of *CpYUC10B* in Chinese cherry fruit reduced abscission at S3 and increased mature fruit set, while its silencing produced the opposite effect. *CpYUC10B* transient overexpression elevated IAA content across Chinese cherry fruit tissues, reduced ABA accumulation in flesh, and suppressed cell wall hydrolase (β-GC and PME) activities in the pedicel. The downregulation of cell wall hydrolase genes (*CpEXLB1*, *CpBGL12*, *CpXTH23*, and *CpPME7*) in overexpressed fruits, coupled with their upregulation in silenced fruits, confirms that *CpYUC10B*-mediated IAA synthesis acts upstream of cell wall remodeling. This finding is consistent with studies on loss-of-function mutants of *YUC* family genes in Arabidopsis and rice, which exhibit typical auxin deficiency phenotypes, including embryonic developmental defects and abnormal organ abscission (Kim et al., 2007; Xu et al., 2020).

Intriguingly, *CpYUC10B* also influenced polar auxin transport capacity. The overexpression of *CpYUC10B* upregulated *CpPIN1* expression level in the flesh, pedicels and embryos, whereas silencing produced inverse expression patterns. This modulation suggests that *CpYUC10B*-derived IAA may feedback-regulate its own distribution via PIN-dependent transport, optimizing the source-sink auxin gradient required for AZ maintenance. This aligns with recent evidence that *YUC*-mediated local auxin biosynthesis and *PIN*-directed polar transport constitute functionally interdependent modules in abscission control (He et al., 2025; Meng et al., 2023; Zhao et al., 2024).

### 4.3 Working model of insufficient synthesis of IAA leading to fruit abscission

**Figure 7:**
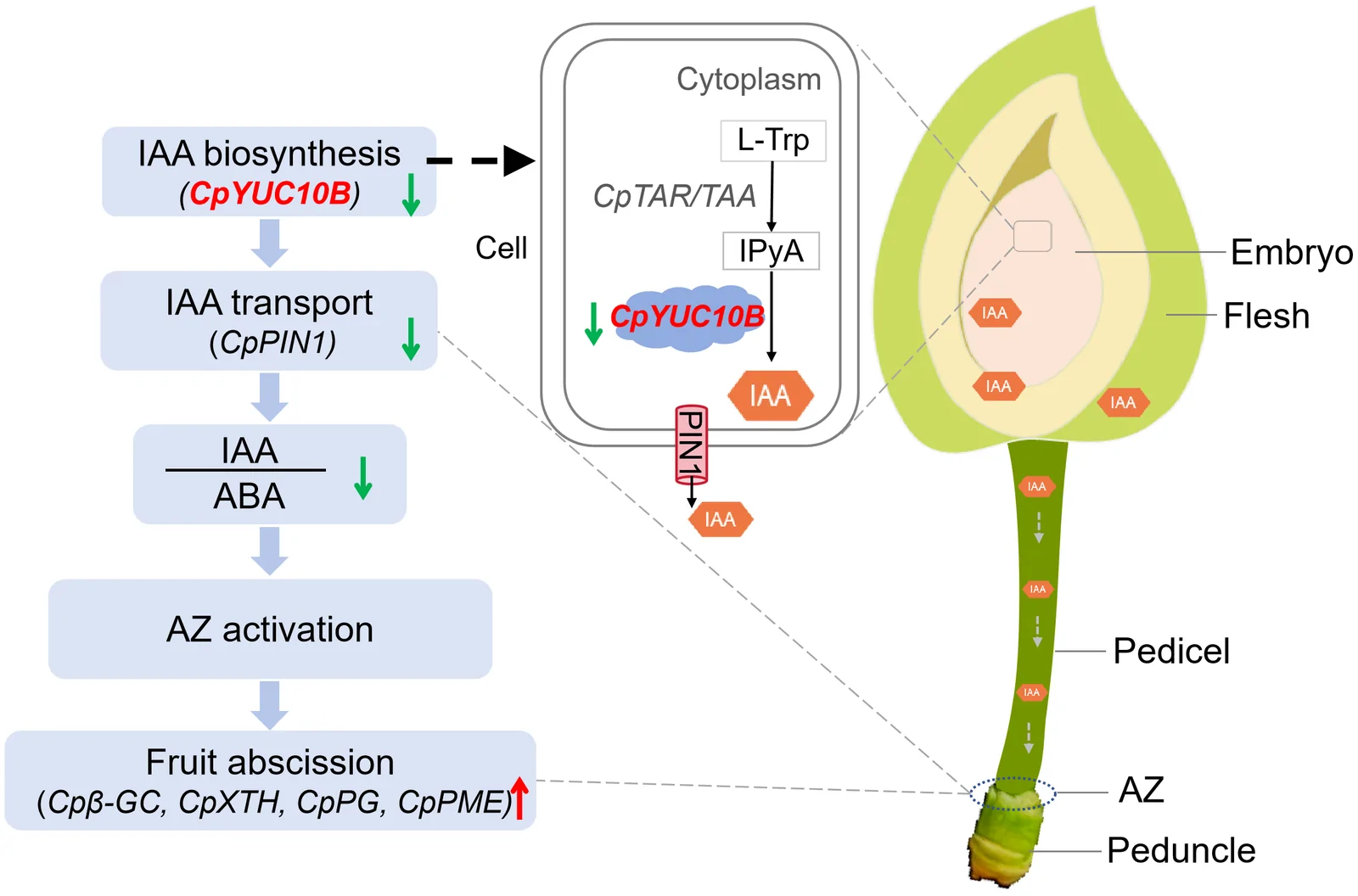
Diagram of *CpYUC10B* involved in auxin-mediated fruit abscission in Chinese cherry. Red and green arrows represent upregulation and downregulation, respectively.

In summary, we propose a working model in which *CpYUC10B*-mediated auxin biosynthesis regulates fruit abscission in Chinese cherry (**Figure 7**). The model involves three key aspects: (i) insufficient auxin biosynthesis: at S3, suppressed *CpYUC10B* expression impairs tryptophan-dependent auxin biosynthesis, depleting embryo IAA; (ii) disrupted polar auxin transport: the resulting low IAA alters expression of the auxin efflux carrier *CpPIN1*, thereby impairing polar transport from the embryo to the abscission zone (AZ); and (iii) AZ cell separation: the collapsed auxin gradient sensitizes AZ cells to abscission signals, activating cell wall hydrolase genes and ultimately triggering fruit abscission. Our study establishes a direct link between auxin biosynthesis and fruit abscission in Chinese cherry, identifying *CpYUC10B* as a key target for reducing premature fruit abscission. The upstream regulatory mechanisms governing *CpYUC10B* expression remain to be elucidated.

## 5 Conclusion

This study demonstrates that massive fruit abscission in large-fruited Chinese cherry LQ during the pit hardening stage results from defective endogenous IAA biosynthesis triggered by abnormal embryo development, thereby establishing a causal relationship between embryo abortion and fruit abscission. Through transient overexpression, VIGS, and stable heterologous transformation in tomato, we verified that *CpYUC10B* encodes a key rate-limiting enzyme governing fruit abscission during the pit hardening stage of Chinese cherry. Overexpression of *CpYUC10B* markedly enhances endogenous IAA biosynthesis, remodels polar auxin transport by upregulating *CpPIN1* expression, suppresses cell wall hydrolase activity in the abscission zone, and ultimately alleviates fruit abscission. Collectively, *CpYUC10B* serves as an excellent target gene for breeding Chinese cherry cultivars with reduced fruit abscission rates.

## Data Availability

The datasets presented in this study can be found in online repositories. The names of the repository/repositories and accession number(s) can be found below: https://db.cngb.org/cnsa/, CNP0008745.
