# Supplementary material for: Auxin biosynthetic key gene CpYUC10B in embryo involved in large-fruited Chinese cherry massive abscission during the pit hardening process
Source: Front Plant Sci. 2026 Aug 18;17:1923869. doi: 10.3389/fpls.2026.1923869 (PMC13529990; doi:10.3389/fpls.2026.1923869)
Supplement: Supplementary file 1 [file DataSheet1.docx]

**Supplementary material**

**Auxin biosynthetic key gene *CpYUC10B* in embryo involved in large-fruited cherry massive abscission during the pit hardening process**

**Authors:**

Hao Wang^1^, Xiaocheng Liu^1^, Quan Su^1^, Yunfan Yang^1^, Xuping Wu^1^, Jing Zhang^1, 2^, Yan Wang^1, 2^, Wen He^1, 2^, Zhiwei Wu^1^, Mengyao Li^1^, Qing Chen^1, 2^, Ya Luo^1^, Yong Zhang^1^, Haoru Tang^1^, Xiaorong Wang^1, 2*^

**Affiliation:**

^1^College of Horticulture, Sichuan Agricultural University, Chengdu 611130, Sichuan, China;

^2^Key Laboratory of Agricultural Bioinformatics (Ministry of Education), Sichuan Agricultural University, Chengdu 611130, Sichuan, China

***Correspondence:**

Xiaorong Wang

wangxr@sicau.edu.cn

**Table S1 List of primers used in this study.**

| **Primer** | **Sequence (5'→3')** | **Usage** |
| --- | --- | --- |
| CpYUC10B-qPCR-F | AGAGAGGCCAACAGAAGGTC | RT-qPCR |
| CpYUC10B-qPCR-R | CTTGAACCCGGTGCAGAAAA | RT-qPCR |
| CpPIN1-qPCR-F | AAAAGGGGCTGCTTGGAATG | RT-qPCR |
| CpPIN1-qPCR-R | GTCTGGGAACTGTTCGGAGA | RT-qPCR |
| CpPG-qPCR-F | CAGCGTGTCACTCTTGGAGT | RT-qPCR |
| CpPG-qPCR-R | GCCGGATGAATGTTCCAAGC | RT-qPCR |
| CpBGL12-qPCR-F | ACCCATTGTCCACTGGTGAC | RT-qPCR |
| CpBGL12-qPCR-R | ACCAAACCGAACAGTGTATCCA | RT-qPCR |
| CpEXLB1-qPCR-F | TGGAAGGACAGTCAACGACG | RT-qPCR |
| CpEXLB1-qPCR-R | CCAGGGATGGCTTTGTTTGC | RT-qPCR |
| CpXTH23-qPCR-F | ATTTTGGTTGTGGCTTCGGC | RT-qPCR |
| CpXTH23-qPCR-R | TGTGGCCCAATCATCTGCAT | RT-qPCR |
| CpPME7-qPCR-F | CGACACCAAGTTCTACAGCG | RT-qPCR |
| CpPME7-qPCR-R | AAAACCCCATCATCCTCCGT | RT-qPCR |
| CpGAPDH-F | TATCCTTTCTGTTTGTGATGAGCCCC | RT-qPCR |
| CpGAPDH-R | CTCGTTGTCGTACCAAGCAATGAC | RT-qPCR |
| CpTIF5A-F | AAGTCTCCACTTCAAAAACTGGCAAGC | RT-qPCR |
| CpTIF5A-R | TATCAATCAGCTGGTAGTCGGTACGG | RT-qPCR |
| Sl-BGL12-F | ATGGAAGCACTGGGGATGTG | RT-qPCR |
| Sl-BGL12-R | TGTCCGATAAAGGCTCAGGC | RT-qPCR |
| Sl-PME7-F | TTCCACCTTCAGAGTCCCCA | RT-qPCR |
| Sl-PME7-R | GCAGCAGTTTCCTCCTTCCT | RT-qPCR |
| Sl-XTH23-F | TCTTTGGACTGACAGCCACA | RT-qPCR |
| Sl-XTH23-R | TTTCGCGGACGTATCTTCCC | RT-qPCR |
| Sl-PG-F | GTACTATCGATGGCCAGGGC | RT-qPCR |
| Sl-PG-R | CAGTGGCCTTGATTCCGACT | RT-qPCR |
| Sl-EXLB1-F | GTTCTCTGCAGCCCACTAT | RT-qPCR |
| Sl-EXLB1-R | ATCACCTGCACCTTGGTCTG | RT-qPCR |
| SlPIN1-F | AATCTAGACCAGCCTGCAGC | RT-qPCR |
| SlPIN1-R | TGGAAGCAGCTGCCATAACA | RT-qPCR |
| SlActin-F | TGTCCCTATTTACGAGGGTTATGC | RT-qPCR |
| SlActin-R | CAGTTAAATCACGACCAGCAAGAT | RT-qPCR |
| SlGAPDH-F | CTGCTCACTTGAAGGGTGGT | RT-qPCR |
| SlGAPDH-R | GACAATGTCCAGCTCTGGCT | RT-qPCR |
| OE-CpYUC10B-F | aaatcaagattaaagggtaccATGGAGCAAGAGAAGGCAGTGA | Overexpressing, subcellular localization |
| OE-CpYUC10B-R | tttgaaggttgtcattctagaTTACTGTAGAAATGACTTGATATCATTTGC |  |
| pTRV2-CpYUC10B-F | gagacgcgtgagctcggtaccGAGAAGGCAGTGATTATAGTCGGG | Gene-silencing |
| pTRV2-CpYUC10B-R | gtgagtaaggttaccgaattcCTCAACATTTCTGTGGTACAGGATG | Gene-silencing |

**Table S2 Detail of genes related to auxin biosynthesis, transport, degradation and signal transduction in turquoise and cyan modules**

| **Gene ID** | **Embryo** | | |  | **Pedicel** | | |  | **Flesh** | | | **Description** |
| --- | --- | --- | --- | --- | --- | --- | --- | --- | --- | --- | --- | --- |
|  | **18N** | **27N** | **27AN** |  | **18N** | **27N** | **27AN** |  | **18N** | **27N** | **27AN** |  |
| Cpseudocerasus_HiC_scaffold_8_0600 | 20.8802 | 0.0809 | 0.0417 |  | 0.3872 | 1.4111 | 0.0000 |  | 9.9977 | 4.9813 | 0.6934 | Tryptophan minotransferase-related protein 4 |
| Cpseudocerasus_HiC_scaffold_8_2784 | 0.0266 | 6.9591 | 6.0731 |  | 3.3501 | 6.9251 | 0.9843 |  | 0.5471 | 8.9591 | 3.5367 | indole-3-pyruvate  monooxygenase YUCCA10 |
| Cpseudocerasus_HiC_scaffold_7_1982 | 120.5717 | 72.3190 | 69.4177 |  | 56.1659 | 34.9111 | 3.7673 |  | 45.7851 | 21.7635 | 17.3980 | 2-oxoglutarate-dependent  dioxygenase DAO1 |
| Cpseudocerasus_HiC_scaffold_5_2410 | 25.6226 | 4.3603 | 2.6370 |  | 47.5497 | 12.4926 | 0.0000 |  | 23.9870 | 0.3091 | 0.0000 | Auxin efflux carrier  component 1 |
| Cpseudocerasus_HiC_scaffold_7_0086 | 10.0409 | 6.9868 | 6.2281 |  | 7.3068 | 11.0775 | 0.1843 |  | 3.3553 | 0.2549 | 0.0070 | Auxin efflux carrier component 1C |
| Cpseudocerasus_HiC_scaffold_2_2946 | 131.3457 | 70.6594 | 14.0388 |  | 51.7318 | 34.5513 | 19.8741 |  | 38.0314 | 2.0843 | 2.2636 | Auxin transporter-like protein 2 |
| Cpseudocerasus_HiC_scaffold_3_2982 | 29.9963 | 5.3775 | 2.8101 |  | 6.2656 | 7.6192 | 5.7804 |  | 10.0419 | 1.9303 | 4.5033 | High affinity nitrate ransporter 2.7 |
| Cpseudocerasus_HiC_scaffold_1_3960 | 334.4368 | 25.0661 | 2.1834 |  | 300.6688 | 106.1378 | 0.0582 |  | 38.8976 | 3.3792 | 0.0415 | Protein Walls Are Thin 1 |
| Cpseudocerasus_HiC_scaffold_1_5092 | 8.9020 | 2.3224 | 1.1551 |  | 4.9626 | 2.4135 | 1.3413 |  | 14.0242 | 2.0552 | 1.3138 | DNA topoisomerase 2-binding protein |
| Cpseudocerasus_HiC_scaffold_1_5921 | 484.7276 | 152.4922 | 106.9267 |  | 44.8762 | 59.8981 | 6.7234 |  | 48.8427 | 8.5950 | 7.9766 | Auxin-responsive protein  IAA8 |
| Cpseudocerasus_HiC_scaffold_6_2195 | 166.5444 | 53.8779 | 47.8384 |  | 60.9999 | 52.3188 | 13.6899 |  | 71.2763 | 11.8467 | 6.5562 | Auxin-responsive protein  IAA9 |
| Cpseudocerasus_HiC_scaffold_1_0489 | 14.8546 | 3.5002 | 4.1564 |  | 3.5919 | 3.3658 | 0.0172 |  | 2.9480 | 0.0200 | 0.0075 | Auxin-responsive protein  IAA13 |
| Cpseudocerasus_HiC_scaffold_3_0754 | 278.3047 | 174.3055 | 68.6574 |  | 26.1522 | 69.6239 | 14.5378 |  | 137.6660 | 38.5757 | 14.0706 | Auxin-responsive protein  IAA17 |
| Cpseudocerasus_HiC_scaffold_4_0490 | 38.9790 | 7.8468 | 5.7267 |  | 31.9757 | 10.3798 | 0.3780 |  | 56.5885 | 12.6285 | 1.0038 | Auxin response factor 3 |
| Cpseudocerasus_HiC_scaffold_6_0948 | 46.4709 | 4.7420 | 1.5278 |  | 14.1732 | 13.5801 | 0.0698 |  | 37.2624 | 4.5413 | 1.2796 | Auxin response factor 4 |
| Cpseudocerasus_HiC_scaffold_5_1425 | 10.6029 | 7.9072 | 4.0959 |  | 0.8396 | 0.6891 | 0.1376 |  | 1.5541 | 0.0652 | 0.1553 | Auxin response factor 9 |
| Cpseudocerasus_HiC_scaffold_2_2402 | 4.9231 | 9.3092 | 3.7542 |  | 4.1187 | 1.8489 | 0.1463 |  | 6.8801 | 1.2687 | 0.5771 | Auxin response factor 18 |
| Cpseudocerasus_HiC_scaffold_7_2034 | 2.3905 | 1.0312 | 1.2615 |  | 4.5237 | 3.2453 | 0.8161 |  | 3.5217 | 0.3841 | 0.1585 | Auxin response factor 19 |
| Cpseudocerasus_HiC_scaffold_7_1777 | 30.4317 | 0.3224 | 12.8063 |  | 0.9160 | 0.1965 | 15.3408 |  | 1.5721 | 0.0000 | 0.2636 | Auxin-responsive protein SAUR50 |

**FIGURE S1** Fruit development pattern of LQ, HF, NH631 and BJ7-2 landraces. **(A)** Fruit phenotypes for five development stages. **(B)** Dynamic changes of fruit weight during fruit development. (S1) The first physiological fruit abscission stage; (S2) pre-fruit pit hardening stage; (S3) fruit pit hardening stage; (S4) fruit color transition and swelling stage; (S5) fruit ripening stage. LQ, HF, and BJ7-2 showed similar developmental durations lasting 51–54 days, with stages S3 to S5 corresponding to DAF 25–36, 37–45, and ≥46 days, respectively. By contrast, NH631 had a shorter developmental period of only 42 days, and its S3~S5 corresponded to DAF 25–33, 34–36, and ≥37 days, respectively.

**FIGURE S2** Observation of stigma morphology and determination of pollen germination rate in LQ. **(A)**, **(B)** and **(C)** illustrated the stigma morphology and anther of Chinese cherry LQ at the bellflower stage and full-bloom stage, respectively. **(D)** Pollen germination in LQ at the full-bloom stage. **(E)** Comparison of pollen germination rates of LQ, HF, BJ7-2 and NH631.

**FIGURE S3** Correlation analysis between fruit abscission rate and embryo abortion rate of LQ, HF, NH631 and BJ7-2 landraces.

### FIGURE S4 Sequence alignment of YUC10 from Chinese cherry, *Arabidopsis thaliana*, pear, rose and strawberry.

### FIGURE S5 Identification and relative expression analysis of *CpYUC10B* transgenic tomato. (A) Identification of transgenic tomato plants overexpressing *CpYUC10B*. (B) Expression level quantification of *CpYUC10B* in transgenic tomato plants.
